# Catalytic Sulfation of Betulin with Sulfamic Acid: Experiment and DFT Calculation

**DOI:** 10.3390/ijms23031602

**Published:** 2022-01-29

**Authors:** Aleksandr S. Kazachenko, Feride Akman, Natalya Yu. Vasilieva, Noureddine Issaoui, Yuriy N. Malyar, Aleksandr A. Kondrasenko, Valentina S. Borovkova, Angelina V. Miroshnikova, Anna S. Kazachenko, Omar Al-Dossary, Marek J. Wojcik, Yaroslava D. Berezhnaya, Evgeniy V. Elsuf’ev

**Affiliations:** 1 Department of Organic and Analytical Chemistry, Institute of Nonferrous Metals and Materials Science, Siberian Federal University, pr. Svobodny 79, 660041 Krasnoyarsk, Russia; vasilyeva.nata@mail.ru (N.Y.V.); yumalyar@gmail.com (Y.N.M.); bing0015@mail.ru (V.S.B.); miroshnikova.av@icct.krasn.ru (A.V.M.); kaalla@list.ru (A.S.K.); 2 Institute of Chemistry and Chemical Technology, Krasnoyarsk Scientific Center, Siberian Branch, Russian Academy of Sciences, Akademgorodok, 50, bld. 24, 660036 Krasnoyarsk, Russia; kondrasenko@icct.ru (A.A.K.); zyppa90298@gmail.com (Y.D.B.); yelsufyevev@gmail.com (E.V.E.); 3 Vocational School of Food, Agriculture and Livestock, University of Bingöl, Bingöl 12000, Turkey; chemakman@gmail.com; 4 Laboratory of Quantum and Statistical Physics (LR18ES18), Faculty of Sciences, University of Monastir, Monastir 5079, Tunisia; issaoui_noureddine@yahoo.fr; 5 Department of Physics and Astronomy, College of Science, King Saud University, P.O. Box 2455, Riyadh 11451, Saudi Arabia; omar@ksu.edu.sa; 6 Faculty of Chemistry, Jagiellonian University, 30-387 Krakow, Poland; wojcik@chemia.uj.edu.pl; 7 Institute of Chemical Technologies, Siberian State University of Science and Technology, pr. Mira 82, 660049 Krasnoyarsk, Russia

**Keywords:** betulin, sulfation, catalysis, density functional theory (DFT), sulfated betulin

## Abstract

Betulin is an important triterpenoid substance isolated from birch bark, which, together with its sulfates, exhibits important bioactive properties. We report on a newly developed method of betulin sulfation with sulfamic acid in pyridine in the presence of an Amberlyst^®^15 solid acid catalyst. It has been shown that this catalyst remains stable when being repeatedly (up to four cycles) used and ensures obtaining of sulfated betulin with a sulfur content of ~10%. The introduction of the sulfate group into the betulin molecule has been proven by Fourier-transform infrared, ultraviolet-visible, and nuclear magnetic resonance spectroscopy. The Fourier-transform infrared (FTIR) spectra contain absorption bands at 1249 and 835–841 cm^−1^; in the UV spectra, the peak intensity decreases; and, in the nuclear magnetic resonance (NMR) spectra, of betulin disulfate, carbons С3 and С28 are completely shifted to the weak-field region (to 88.21 and 67.32 ppm, respectively) with respect to betulin. Using the potentiometric titration method, the product of acidity constants K_1_ and K_2_ of a solution of the betulin disulfate H^+^ form has been found to be 3.86 × 10^–6^ ± 0.004. It has been demonstrated by the thermal analysis that betulin and the betulin disulfate sodium salt are stable at temperatures of up to 240 and 220 °C, respectively. The density functional theory method has been used to obtain data on the most stable conformations, molecular electrostatic potential, frontier molecular orbitals, and mulliken atomic charges of betulin and betulin disulfate and to calculate the spectral characteristics of initial and sulfated betulin, which agree well with the experimental data.

## 1. Introduction

Plant biomass is an important feedstock for a wide range of valuable chemicals [1,2,3,4,5]. Catalytic processing of plant lignocellulosic biomass is an urgent task [6]. Birch biomass can serve as a source of various extractive substances. Birch bark is characterized by a particularly high content of extractive substances, which include mono- and triterpenoids, hydrocarbons, alcohols, fatty and resin acids, and phenolic compounds [7,8,9]

Triterpene compounds represent the most important class of bioactive substances promising for use as pharmaceutical active ingredients, drugs, and phytopreparations [10,11,12,13,14,15,16,17]. The betulin derivatives hold a special place in a triterpenoid series. A rich source of betulin is the Betulaceae family, especially Betula alba, Betula pubescens, Betula platyphylla, and Betula pendula [12,14,18]. The betulin content in the birch outer bark ranges within 10–35%, depending on a type of birch, an area and conditions of its growth, an age of the tree, and other factors [15,18]. In the in vitro and in vivo experiments, betulin and its derivatives exhibit the anti-inflammatory, anticonvulsant, antibacterial, antiviral, anti-HIV, antitumor, and other types of biological activity [11,12,13,14,15,16,17,19,20,21,22,23].

Owing to its availability and bioactivity, betulin is well-known as a valuable natural substance, both in its native state and in various modifications. Meanwhile, the low solubility of betulin in water limits its application in medicine, cosmetics, etc. To enhance the water solubility of triterpenoids, different methods are used, including the salt formation, the use of special dosage forms, which ensure the vector delivery of poorly soluble compounds, and nano- and biotechnological techniques [13,16,17,19,20,24,25]. The solubility of triterpenoids can be increased via their chemical modification. In particular, the solubility and bioavailability of triterpenoids can be improved by the complexation with γ-cyclodextrin and other compounds that can form inclusion complexes via the hydrophobic binding [13,17,19,20,24,25,26,27]. Sulfation of betulin improves its water solubility. Sulfuric esters of betulin and betulinic acid were shown to be bioactive [27,28].

The triterpenoid sulfation methods proposed previously [28,29] are based on the use of sulfuric acid and complexes resulting from the interaction of sulfuric anhydride with pyridine or dimethyl sulfoxide. Thus, the synthesis of betulin disulfate and betulinic acid 3-sulfate is carried out via sulfation of betulin and betulinic acid with sulfuric acid in pyridine in the presence of acetic anhydride [28].

Sulfation of triterpenoids with chlorosulfonic or sulfamic acid in an environment of weak bases, e.g., dimethylformamide or dioxane, not only can be accompanied by isomerization of initial betulin [23], but the sulfated triterpenoid can be formed initially in a slightly stable H-form [30]. As is known, the sulfur trioxide pyridine complex is a milder sulfating reagent than the SO_3_–1,4-dioxane complex; it attacks exclusively alcohol groups and does not affect the double bond [31]. The use of pyridine in betulin sulfation instead of 1,4-dioxane and N, *N*-dimethylformamide excludes the formation of sulfated betulin in a slightly stable H-form [30]; sulfated betulin has a form of a stable salt.

The aim of this work was to develop a new method for sulfation of betulin with sulfamic acid in the presence of an Amberlyst^®^15 solid acid catalyst and to examine the reaction products by Fourier-transform infrared ultraviolet-visible (UV-Vis), and nuclear magnetic resonance spectroscopy, thermogravimetric analysis, and X-ray diffraction (XRD) techniques. In addition, the results obtained were theoretically confirmed by the density functional theory method.

## 2. Results

Sulfamic acid is often used for sulfation of natural compounds [32,33,34]. In the absence of activators, it exhibits a weak activity in the sulfation reactions [35].

In [33,34,36,37], several activators of the process of sulfation of natural polymers with sulfamic acid were proposed: 1,4-dioxane, urea, N, *N*-dimethylformamide, morpholine, piperidine, and pyridine. In [35,38,39], urea-based activators of sulfation with sulfamic acid were studied. It was shown that urea has the highest activity. In addition, urea as an activator of the sulfation of components of natural organic raw materials with sulfamic acid was investigated [40,41,42,43,44,45,46,47]. It should be noted that the activating ability of urea and its derivatives in the reactions of sulfation with sulfamic acid can be related to the presence of hydrogen bonds and their number [48,49,50,51].

The main drawback of the above-mentioned sulfation methods is that the activator cannot be isolated from the reaction mixture for further reuse. In addition, the use of urea as an activator can cause side carbamation reactions [43,44,52], the role of which remains unclear.

In this work, we studied the possibility of betulin sulfation with sulfamic acid in pyridine in the presence of an Amberlyst^®^15 solid catalyst. The data on the effect of the time of betulin sulfation with sulfamic acid on the sulfur content in the reaction product are given in Table 1. It can be seen that the sulfur content in sulfated betulin increases with the process time.

In addition, the stability of the catalyst during its repeated use in the process of sulfation of betulin with sulfamic acid was examined. It is noteworthy that the sulfur content in betulin sulfate slightly decreases at the catalyst reuse.

Thus, we can judge about the stability of an Amberlyst-15^®^ catalyst during the catalytic sulfation of betulin with sulfamic acid.

Based on the experimental data obtained, we proposed a scheme for the catalytic sulfation of betulin with sulfamic acid:

R-SO_3_H + SO_3_∙NH_3_ → R-SO_3_-NH_4_^+^ + SO_3_^−^ (1)

SO_3_ + pyridine→ SO_3_ * pyridine (2)

SO_3_ * pyridine (sulfating complex) + R’-(OH)_2_ → R’-(O-SO_3_H)_2_ + pyridine (3)

R’-(O-SO_3_H)_2_ + R-SO_3_NH_4_ → R’-(O-SO_3_ NH_4_)_2_ + R-SO_3_H, (4)

where R is the catalyst matrix and R’ is the betulin molecule.

It was shown in [53] that sulfamic acid can be in the zwitterionic form and, in the presence of organic bases, can form a donor–acceptor complex, which will be more active in the sulfation reactions [54,55].

According to the scheme proposed by us, during the catalytic sulfation of betulin, first, the sorption of the zwitterionic form of sulfamic acid on the catalyst matrix occurs, which is followed by decomposition of the acid into sulfur trioxide and ammonia, and sulfur trioxide interacts with 1,4-dioxane with the formation of a sulfating complex. The sulfating complex sulfates betulin and removes sulfur trioxide. This is followed by the exchange by ammonium cations between the catalyst matrix and the acidic form of betulin sulfate.

### 2.1. Fourier-Transform Infrared Spectroscopy Study

**Figure 1:**
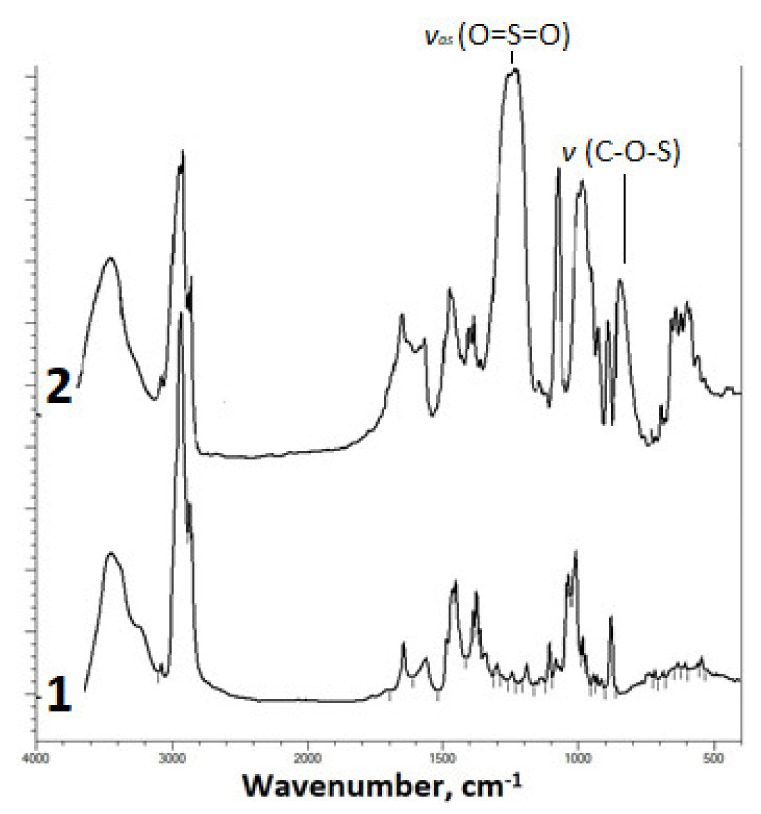
FTIR spectra of (**1**) betulin and (**2**) sulfated betulin in the sodium form.

The introduction of the sulfate group into the betulin molecule was confirmed by IR spectroscopy (Figure 1).

The FTIR spectrum of the betulin disulfate sodium salt contains, along with the absorption bands characteristic of the initial betulin, a high-intensity band of asymmetric stretching vibrations υ_as_ (O=S=O) at 1249 cm^−1^ and an absorption band of stretching vibrations υ (C–O–S) [56,57] in the range of 835–841 cm^−1^.

### 2.2. Ultraviolet—Visible Spectroscopy Study

**Figure 2:**
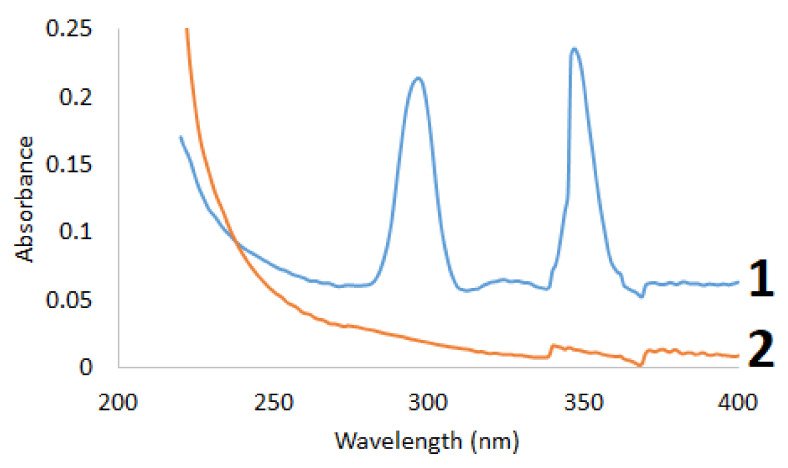
UV spectra of (**1**) betulin and (**2**) sulfated betulin.

The initial and sulfated betulin was examined by UV-Vis spectroscopy (Figure 2).

To qualitatively identify the presence of sulfo groups in the betulin sulfation products, the electronic absorption spectra in the range of 220–400 nm were reproduced in the alcohol solution. It can be seen in Figure 2 that the UV spectra of the initial and sulfated betulin have different profiles, which is indicative of the presence of new functional groups in the sulfated products [58]. A factor indicating the introduction of a sulfo group into the betulin structure is a decrease in the total intensity of the sulfation products. The introduction of an additional functional group with an increase in the average molecular weight leads to a decrease in the intensity in the UV range.

### 2.3. Nuclear Magnetic Resonance Study

The composition and structure of the betulin disulfate sodium salt was confirmed by ^13^C NMR spectroscopy. According to the literature data [59], the chemical shift of the secondary C3 carbon atom bonded to the hydroxyl group is observed at 78–79 ppm and the chemical shift of the primary C28 carbon atom, at 59–60 ppm. An analysis of the ^13^C NMR spectra of the initial betulin and the betulin disulfate sodium salt showed that the chemical shift of the C3 carbon atom in the initial betulin is observed at 78.25 ppm and the chemical shift of the C28 carbon atom, at 58.96 ppm. In the synthesized betulin disulfate, the chemical shifts of the С3 and С28 carbons in comparison with betulin are completely shifted to the low-field region (to 88.21 and 67.32 ppm, respectively). This proves the complete replacement of the betulin hydroxyl groups by the sulfate.

### 2.4. X-Ray Diffraction Study

**Figure 3:**
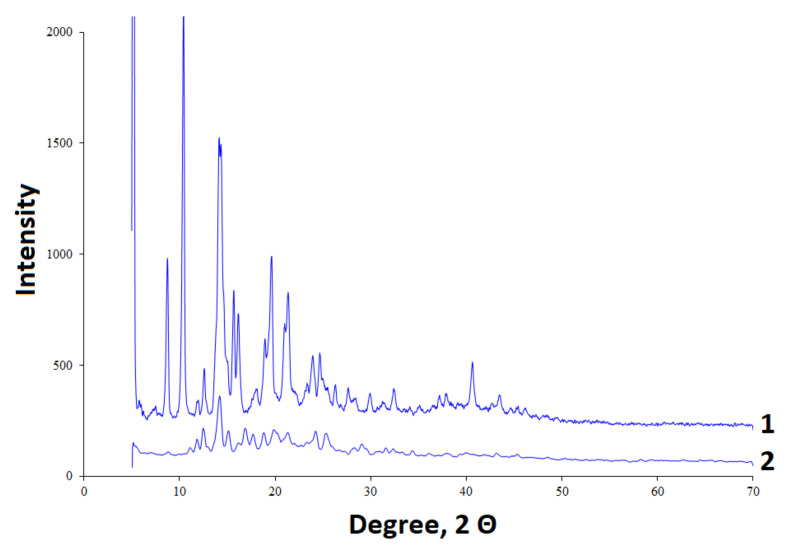
XRD diffraction patterns of (**1**) betulin and (**2**) betulin sulfate.

The initial betulin and betulin sulfate were analyzed by X-ray diffractometry (Figure 3). The initial betulin has a crystalline structure with the high-intensity bands [60]. During sulfation, a decrease in the crystallinity of betulin is observed, as is the case with its chemical modification by other methods [61].

### 2.5. Thermal Analysis

**Figure 4:**
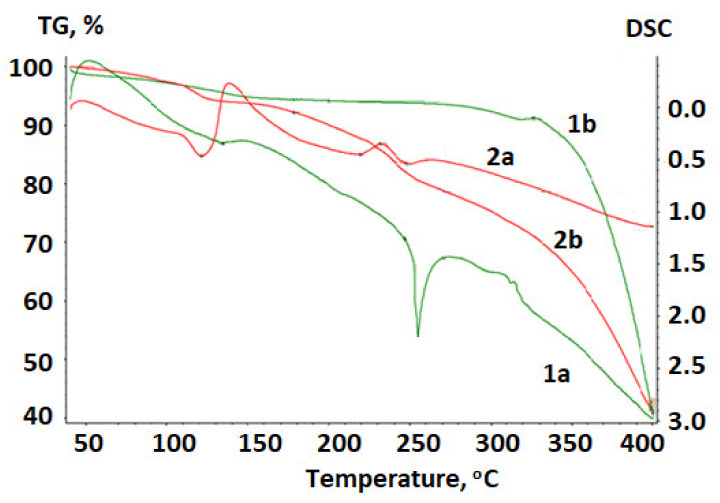
Thermogram of (**1**) betulin and (**2**) betulin disulfate sodium salt: (**a**) DTA and (**b**) TG curves.

Figure 4 shows a thermogram of betulin and the betulin disulfate sodium salt obtained upon heating in the argon atmosphere.

The temperature dependence of the weight loss (the TG curve) for betulin has a plateau; the horizontal section is indicative of the stability of the chemical compound in the investigated temperature range at the absence of chemical transformations. A vertical step in the curve is indicative of the chemical decomposition of the material [62].

At a temperature of 134.5 °C, a loss of water contained in betulin is observed. The weight loss is 5.83%. The TG curve reflects an intense loss in the sample mass above 260 °C.

To determine the transformation temperatures more accurately, a differential notation was used. A peak in the DTA curve in the range of 240–260 °C is indicative of a phase transformation in betulin, which is accompanied by the endothermic effect. This is a first-order phase transition; in this region, betulin melts with the subsequent decomposition.

In contrast to initial betulin, in the betulin disulfate sodium salt the water loss occurs earlier and the weight loss is 7.76%.

In the DTA curve at temperatures of 220–230 °C, the exothermic effect is reflected, which corresponds to the decomposition of the betulin disulfate sodium salt, apparently with the SO_2_ release. The weight loss is 15.5%, according to the sulfur content in betulin disulfate. The degree of decomposition of betulin disulfate with the SO_2_ release in this region is 79%, which is consistent with the data reported in [43,55,63]. We can state that betulin and the betulin disulfate sodium salt are stable at temperatures of up to 240 and 220 °C, respectively.

### 2.6. Acidity Constants

**Figure 5:**
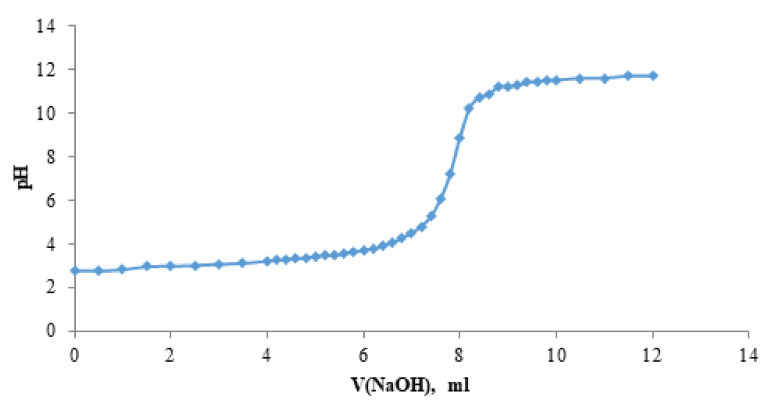
Dependence of pH of the solution of the H + form of betulin disulfate on the sodium hydroxide volume.

In this work, the product of acidity constants K_1_ and K_2_ of the solution of the betulin disulfate H ^+^ form was determined by a potentiometric titration (Figure 5)**.** The average product of the first and second dissociation constants K_1_ and K_2_ was found to be 3.86 × 10^−6^ ± 0.004. Figure 5 shows the dependence of pH of the solution of the betulin disulfate H ^+^ form on the sodium hydroxide volume.

Since the titration curve contains only one jump, it is obvious that the H ^+^ form of betulin disulfate has similar values of the first and second dissociation constants K_1_ and K_2_. This, most likely, originates from the betulin disulfate structure, in which sulfate groups are distant from each other.

This conclusion does not contradict the data on dissociation constants of the known dicarboxylic acids with carboxyl groups significantly distant from each other [64]; therefore, for example, in adipic acid, the K_1_ and K_2_ values are of the same order of magnitude: 3.7 × 10^−5^ and 1.93 × 10^−5^, respectively.

According to the obtained acidity constant, the H ^+^ form of betulin disulfate is an acid stronger than carboxylic acids, but weaker than sulfonic acids. Basing on the determined constant, the H + form of betulin disulfate was confirmed, which can be obtained by adding a mineral acid salt to the aqueous solution.

### 2.7. Theoretical Calculations

#### 2.7.1. Optimized Geometry and MEP Analysis of Betulin and Betulin Disulfate

**Figure 6:**
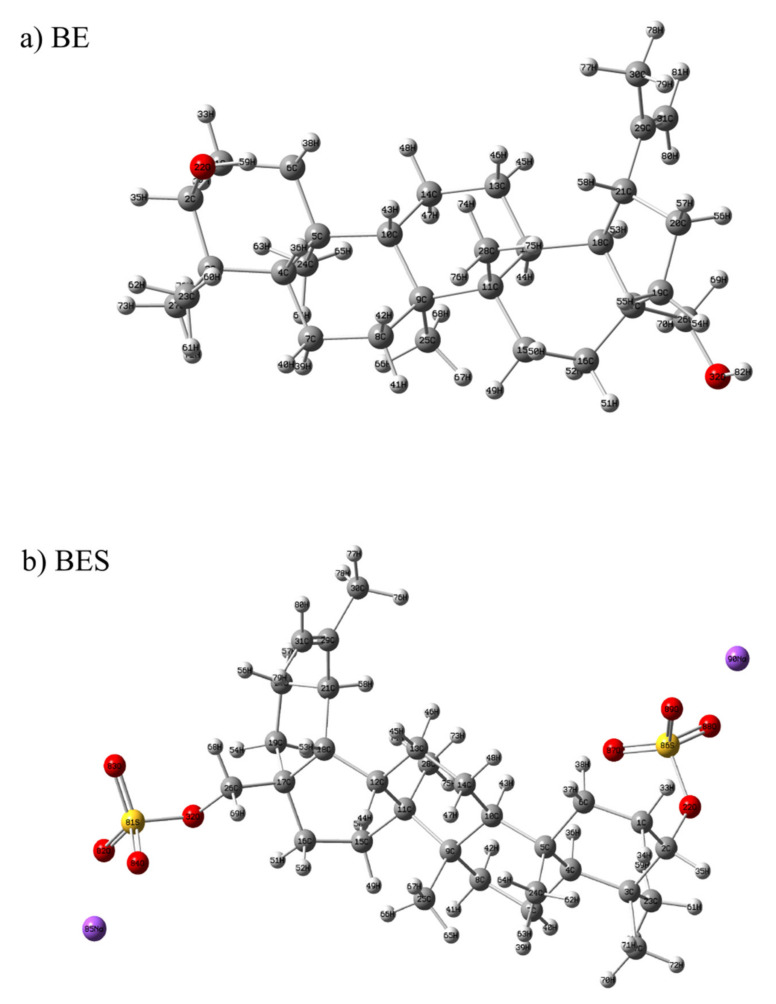
Geometrical structures of (**a**) betulin and (**b**) sulfated betulin optimized by the CAM-B3LYP/6-31 + G(d, p) level of theory.

The primary task of the quantum chemical calculations is to determine the optimized geometry of a molecule [65,66,67]. The optimized geometry of betulin and betulin disulfate was calculated using the CAM-B3LYP/6-31 + G(d, p) method. The data obtained are presented in Figure 6, where BE is betulin and BES is betulin disulfate.

**Figure 7:**
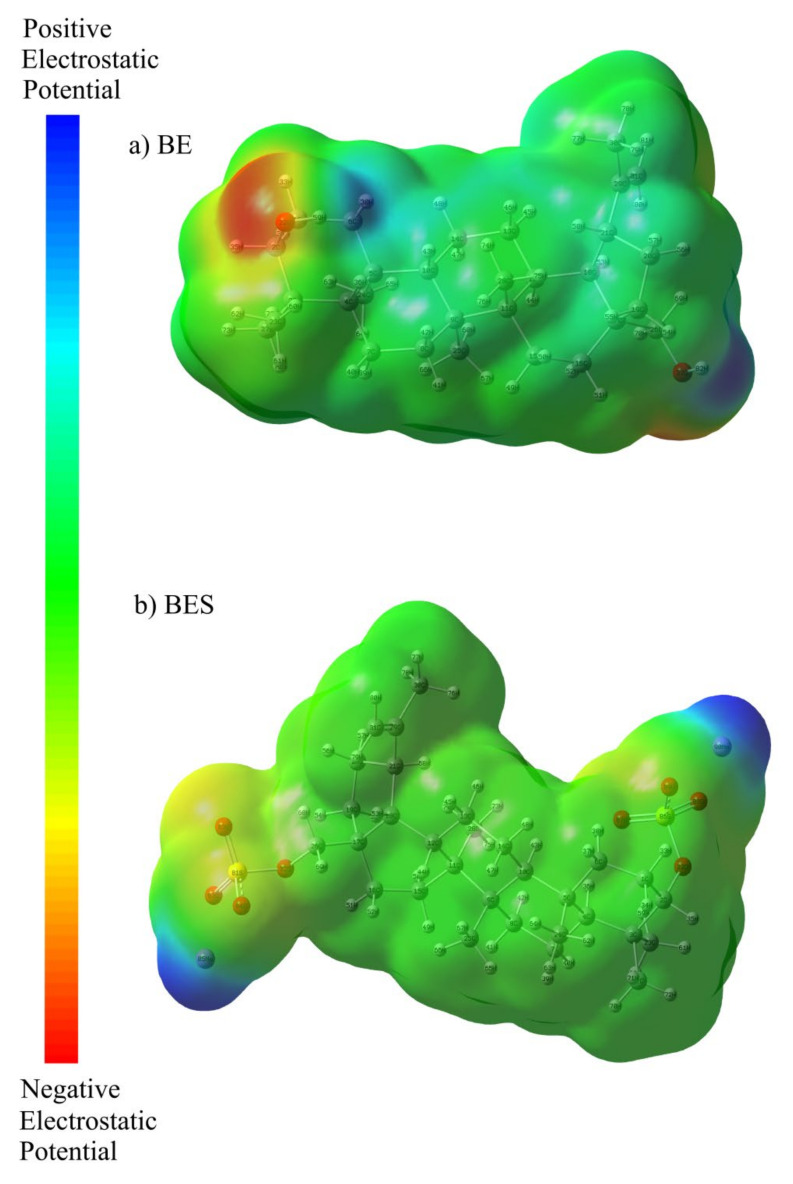
MEP surfaces of (**a**) betulin and (**b**) sulfated betulin built using the CAM-B3LYP/6-31 + G (d, p) level of theory.

To estimate the positions of electrophilic and nucleophilic actions, as well as interactions of hydrogen bonds, the MEP was calculated, which, in turn, is related to the electron density and extremely useful [68,69]. For this purpose, MEP maps of betulin and betulin disulfate were built, which helped us to estimate the regions of nucleophilic and electrophilic attacks and the interaction of hydrogen bonds. The MEP surfaces of betulin and disulfated betulin were determined by the CAM-B3LYP/6-31+G(d, p) method used for optimizing molecules. The three-dimensional surface maps are shown in Figure 7.

In the MEP analysis, the reactive regions are marked by different color codes corresponding to the electrostatic potential color order, e. g., red <orange <yellow <green <blue. In the MEP maps, the blue color indicates an electron-deficient area, which has a positive electrostatic potential, and the red color indicates an electron-rich area, which has a negative electrostatic potential. The green color in the MEP maps corresponds to a neutral region with zero electrostatic potential [44,70].

As can be seen in Figure 7a, hydrogen atoms attached to oxygen atoms have the lowest electron density and are colored in blue in the map, while the electron density of oxygen atoms is higher, which is reflected by the red color. For betulin disulfate, the picture is different. After the introduction of sulfate groups in the 3 and 28 betulin sites and their stabilization with sodium cations, the MEP maps change. In Figure 7b, the blue color in the betulin disulfate MEP maps is observed mainly above the sodium atoms. In addition, the hydrogen atoms attached to oxygen in the betulin molecule change for the sulfate, the electron density on the oxygen atoms decreases, and the red color changes for yellow and greenish-yellow. A similar phenomenon was observed previously in [40,41,44].

#### 2.7.2. HOMO—LUMO Analysis and Calculated Electronic Properties

The highest occupied molecular orbital (HOMO) and the lowest unoccupied molecular orbital (LUMO) and their respective energies E_HOMO_ and E_LUMO_ are important for obtaining data on the structure and reactivity of substances. The HOMO and LUMO are called the frontier molecular orbital (FMOs) because they can determine the interaction of a molecule with other species.

**Figure 8:**
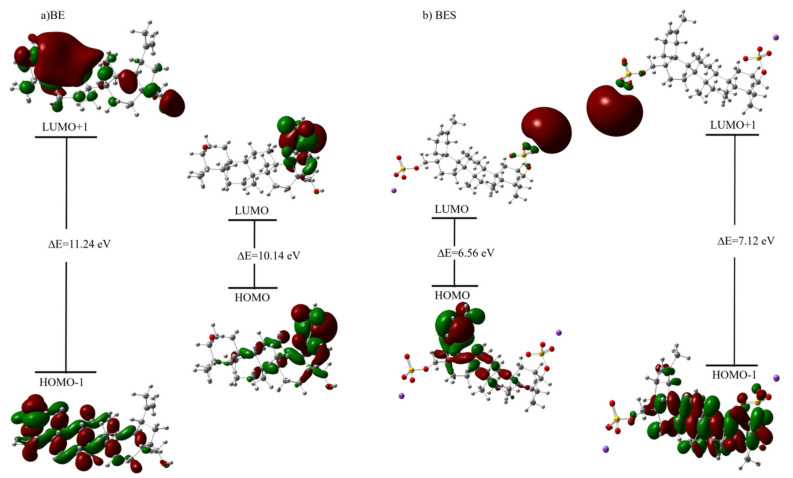
Molecular orbital diagrams of (**a**) betulin and (**b**) sulfated betulin built using the CAM-B3LYP/6-31 + G (d, p) level of theory.

The HOMO is defined as a nucleophile that donates an electron (donor) and the LUMO, as an electrophile that receives an electron from a nucleophile (acceptor) [69,71,72]. A molecule with a narrow band gap suggests the high polarization and is related mainly to the high chemical reactivity and low kinetic stability. The HOMO and LUMO of betulin and betulin disulfate calculated by the CAM-B3LYP/6-31 + G (d, p) method are plotted in Figure 8a and Figure 8b, respectively.

It can be seen in Figure 8 that, during betulin sulfation, the energy gap narrows by 3.5 eV on average, which may be indicative of a higher reactivity of betulin disulfate in comparison with the initial botulin

Basing on the HOMO–LUMO energy gap, nucleophilic index (N), optical softness (σ˳), electronegativity (χ), electron affinity (A), chemical potential (μ), ionization energy (I), hardness (η), softness(ς), electrophilicity index (ω), and maximum charge transfer index (ΔN_max_) were calculated (Table 2) [73,74,75] using the equations

(5) $$I=-E_{HOMO}$$

(6) $$A=-E_{LUMO}$$

(7) χ=−12ELUMO+EHOMO

(8) μ=12ELUMO+EHOMO

(9) η=12ELUMO−EHOMO

(10) $$\zeta =\frac{1}{\eta }$$

(11) $$\omega =\frac{\mu^{2}}{2\eta }$$

(12) $$\Delta \mathrm{Nmax}=-\frac{\mu }{\eta }$$

(13) $$N=\frac{1}{\omega }$$

(14) $$\sigma ˳=\frac{1}{E_{g}}$$

The low kinetic stability and high biological activity, polarizability, and chemical reactivity are indicated by the narrow HOMO–LUMO band gap [76].

The negative values of the chemical potential of molecules suggest that these molecules are stable. The electrophilic index provides information on the ability of a molecule to bind to biomolecules [77]. According to [78], weak electrophiles have an electrophilicity index of ω < 0.8 eV; moderate electrophiles, 0.8 < ω < 1.5 eV; and strong electrophiles, ω > 1.5 eV. In the betulin sulfation process, the electrophilic effect is enhanced, as indicated by an increase in the electrophilicity index from 0.8808 to 2.8626 eV in the CAM-B3LYP method and from 1.2234 to 3.5734 eV in the B3LYP method.

It should be noted that, in the CAM-B3LYP and B3LYP calculations, the introduction of sulfate groups into the betulin molecule leads to a decrease in the chemical potential μ from −2.9897 to −4.3337 eV and from −2.9471 to −3.9148 eV; in the ionization energy I from 8.0636 to 7.6140 eV and from 6.4970 to 6.0592 eV; in the chemical hardness η from 5.0738 to 3.2803 eV and from 3.5499 to 2.1444 eV; in the nucleophilic index N from 1.1353 to 0.3493 eV and from 0.8174 to 0.2798 eV; as well as to an increase in softness ς from 0.1971 to 0.3048 eV and from 0.2817 to 0.4663 eV; in the electron affinity EA from −2.0841 to 1.0534 eV and from −0.6027 to 1.7704 eV; in the electronegativity χ from 2.9897 to 4.3337 eV and from 2.9471 to 3.9148 eV; in the maximum charge transfer index ΔN_max_ from 0.5892 to 1.3211 eV and from 0.8302 to 1.8256 eV; and in the optical softness σ˳ from 0.0985 to 0.1524 eV and from 0.1409 to 0.2332 eV, respectively (Table 2).

#### 2.7.3. Mulliken Atomic Charges

The Mulliken atomic charges play an important role in the use of quantum-chemical calculations as applied to a molecular system for determining its dipole moment, electronic structure, molecular polarizability, atomic charge effect, and many other characteristics [70,79,80]. These charges are expected to affect the electronic parameters, refraction, dipole moment, and polarizability. The Mulliken atomic charges of the initial betulin and its sulfate in the B3LYP/6–31G + (d, p) and CAM-B3LYP/6–31G + (d, p) methods are given in Table 3. The positive charges are localized on sulfur and hydrogen atoms and the negative ones, on oxygen; for carbon atoms there are both the positive and negative charges.

It should be noted that the introduction of a sulfate group into the betulin molecule affects almost all Mulliken atomic charges of all atoms in the system. Therefore, in the CAM-B3LYP and B3LYP calculations, the Mulliken atomic charges for C2 and C26 decrease from 0.18481 to 0.18324 e and increase from 0.18906 to 0.18934, respectively, as was confirmed in [40,41], which, in turn, is related to the electrophilic–nucleophilic effects [81,82,83].

#### 2.7.4. Spectroscopic Analysis

The physicochemical characteristics of natural compounds can be successfully determined by the DFT method [84,85,86,87]. An important aspect of the theoretical spectroscopic studies is obtaining FTIR and NMR spectroscopy data [88,89,90,91,92,93].

**Figure 9:**
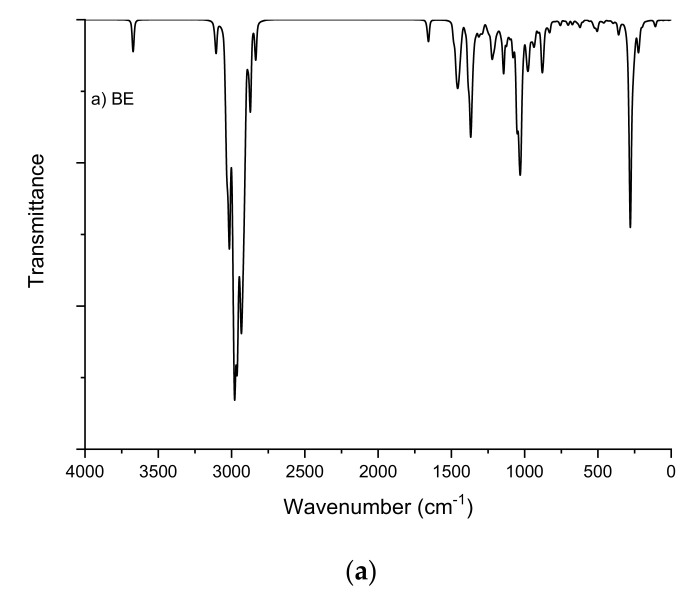

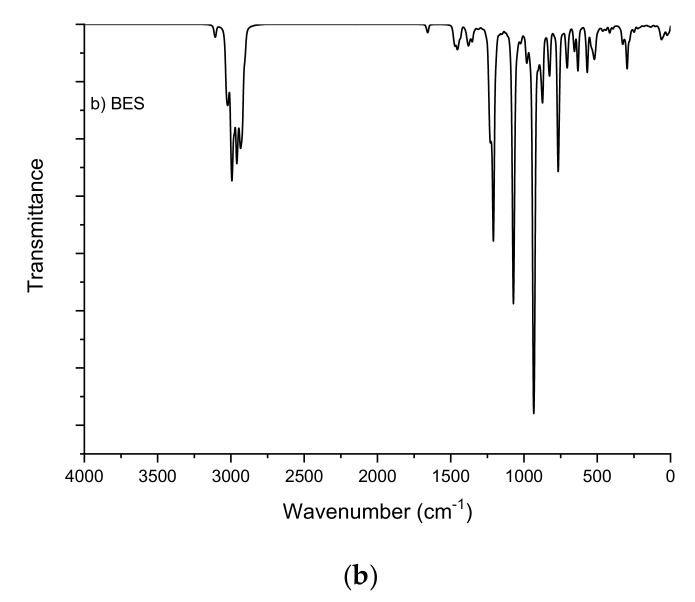
Theoretical FTIR spectra at B3LYP/6-31 + G (d, p) scaled by 0.9608 cm^−1^ for (**a**) betulin and (**b**) betulin sulfate.

To carry out thorough spectroscopic investigations of the initial and sulfated betulin, we calculated theoretical FTIR and NMR spectra. The theoretical FTIR spectra of the initial and sulfated betulin contain several absorption bands with different relative intensities. The spectra were calculated for the DFT method with the 6-31+G (d, p) basis set (Figure 9, Table 4).

#### 2.7.5. O–H Vibration

It is well-known [94,95] that vibrations of the O–H group are most sensitive to the environment. The presence of a hydrogen bond in such a compound allows one to shift to the low-frequency regions with an increase in the intensity in the FTIR spectra. In the theoretical FTIR spectra of the initial betulin, stretching vibrations of OH groups were observed around 3671 and 3670 cm^−1^. In sulfated betulin, these fluctuations were not observed due to the complete replacement of the hydroxyl group by the sulfate group.

#### 2.7.6. C–H Vibration

The heterocyclic CH group gives rise to several fundamental frequencies, including stretching (symmetric and asymmetric in-plane and out-of-plane bending vibrations). The stretching modes (asymmetric and symmetric) usually appear at ~3100 and 3000 cm^−1^ [96,97]. In the theoretical FTIR spectra, vibrations of the C–H group in the betulin samples were observed at 3107, 3032, and 3038–2835 cm^−1^. For betulin sulfate, vibrations of C–H groups are observed at 3107 and 3032, 3033–2901, and 2984 cm^−1^.

#### 2.7.7. C=C Vibration

In the theoretical FTIR spectra, stretching vibrations of the C=C group for betulin and betulin sulfate were observed at 1657 and 1658 cm^−1^, respectively.

#### 2.7.8. O–S Vibration

In the theoretical FTIR spectra, stretching vibrations of the O–S group for sulfated betulin was observed at 1227, 1209, 1074, 1068, 941, and 931 cm^−1^. In the experimental FTIR spectra, vibrations of this group were observed around 1249 cm^−1^ and 835–841 cm^−1^, which is consistent with the results reported in [56,98].

**Figure 10:**
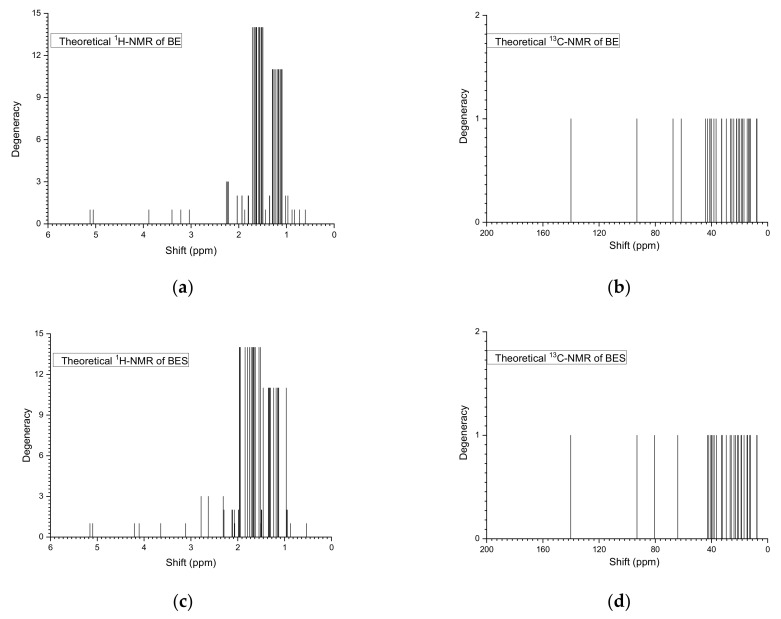
Theoretical ^1^H and ^13^C NMR spectra of betulin (BE) and sulfated betulin (BES) at B3LYP/6-31 + G (d, p).

**Figure 11:**
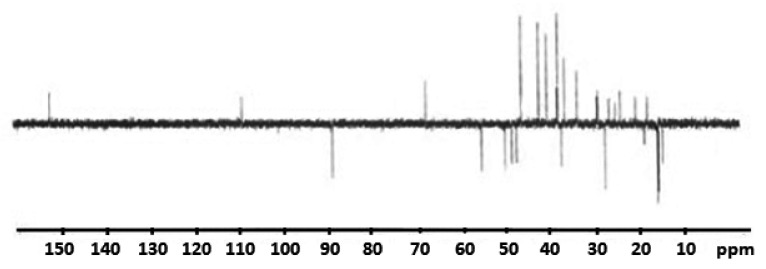
NMR spectrum of the betulin disulfate sodium salt.

In addition, to estimate the qualitative changes in betulin during sulfation, theoretical NMR spectra were calculated (Figure 10, Table 5). According to the data given in Table 5, a strong signal shift from 67.3998 to 80.5108 ppm for the C3 atom (C2 in the table) and from 61.5732 to 63.9693 ppm for the C28 atom (C26 in the table) is observed. The results obtained agree well with the experimental data presented in Figure 11. The difference in numbering is explained by the different numbering in the classical nomenclature of the chemistry of tripertinoid compounds and in software.

## 3. Material and Methods

### 3.1. Sulfation of Betulin

In the sulfation experiment, 50 mL of dehydrated pyridine, 6 g of sulfamic acid, and 2 g of betulin were placed in a 100 mL three-necked flask equipped with a mechanical stirrer and a thermostat. The reaction mass was heated to 90 °C, 0.3 g of an Amberlyst^®^15 catalyst was added, and the mixture was kept at this temperature for 0.5–2.5 h. Then, the reaction mixture was cooled down to 10–15 °C, diluted with 30 mL of water, and neutralized with the 10% aqueous sodium hydroxide solution to pH 9; the catalyst solution was separated by filtration on a paper filter. The solution was evaporated to dryness under a water jet vacuum pump. The solid residue was added with anhydrous ethanol (70 mL) and the mixture was refluxed for 30 min. The hot mixture was filtered and the mother liquor was evaporated to dryness under a water jet vacuum pump. In the physicochemical investigations, betulin disulfate recrystallized from ethanol with the maximum sulfur content was used.

The betulin disulfate H-form was obtained from the betulin disulfate sodium salt by the ionic method on a KU-2-8 cation exchanger according with the procedures described in [56,99,100]. Previously, a commercial KU-2-8 ion exchange resin in the Na^+^ form was transferred to the H^+^ form. To do this, an aqueous 2 M HCl solution was transmitted through a layer of KU-2-8 resin in the Na+ form homogeneously mixed with distilled water and placed in a 50 mL vertical glass column 15–20 mm in diameter with a tap at the bottom. The hydrochloric acid solution flew out of the column. Then, the resin was washed with distilled water until the wash water was neutral in methyl red. The solution of ~1.0 g of betulin disulfate sodium salt recrystallized from ethanol in 50 mL of distilled water was transmitted through the prepared cation resin layer. After the passage of the solution of betulin disulfate sodium salt through the column, the resin in the column was washed three times with distilled water (20 mL each). The washing liquids were collected and the solution was evaporated to dryness under a water jet vacuum pump at a distillation temperature of no higher than 40 °C.

The content of hydrogen ions in the H + form was determined by an acid–base titration. A sample weight of the H^+^ form (0.0400–0.0500 g) was dissolved in 25 mL of water. The 5 mL samples were taken and the titration with 0.01 N sodium hydroxide solution in methyl red until the yellow solution was performed.

The acidity constant of the solution of the betulin disulfate H ^+^ form was determined by a potentiometric titration. A sample weight of the H ^+^ form (0.0400–0.0500 g) was dissolved in a water–alcohol solution consisting of 10 mL of water and 15 mL of ethanol. Three 20 mL aliquots were taken and the potentiometric titration of each of them with the 0.01 N sodium hydroxide solution was performed. The dependence of the solution pH on the titrant volume was plotted. The average value of the acidity constant was calculated using the formula

(15) $$K=\frac{0.5\times A^{3}}{B-\frac{A}{2}}$$

where A is the concentration of hydrogen ions at the equivalent point (mol/L) and B is the initial solution concentration (mol/L).

### 3.2. Methods for Physicochemical Analysis

The elemental analysis of the sulfated samples was carried out on a Thermo Quest Flash EA-1112 elemental analyzer (Italy).

The FTIR spectra of the initial and sulfated betulin were recorded on a Shimadzu IR Tracer-100 spectrometer (Japan) in the wavelength range of 400–4000 cm^−1^. The spectral data were analyzed with the OPUS program (version 5.0). Solid samples for the analysis were prepared in the form of tablets in a KBr matrix (2-mg sample/1000-mg KBr).

The UV-Vis spectra were recorded on a Leki Instruments SS2109-UV scanning spectrophotometer (Finland) using 1-cm quartz cells. Cell thermostating (±0.1 K) was performed in a Haake K15 thermostat connected to a Haake DC10 controller. The absorbance of the process solutions was measured within 220–400 nm. All the measurements were performed at a temperature of 298 K.

The ^13^C NMR spectra were recorded on a Bruker Avance III 600 MHz spectrometer in deuteromethanol with a reference to the deuterium resonance of the solvent.

The X-ray diffraction study was carried out on a DRON-3 X-ray diffractometer (monochromatic Cu*K*_α_ radiation, λ = 0.154 nm) at a voltage of 30 kV and a current of 25 mA. The scanning step was 0.02 deg and the intervals were 1 s per data point. The measurements were performed at the 2Θ Bragg angles ranging from 5.00 to 70.00 Θ.

The thermal analysis was carried out in a corundum crucible using an STA 449 F1 Jupiter instrument (NETZSCH) in the temperature range from 30 to 600 °C at a heating rate of 10 °C/min in an argon flow; the shielding and purge gas flow rates were 20 and 50 mL/min, respectively. The measured data were processed using the NETZSCH Proteus Thermal Analysis 5.1.0 software package supplied with the instrument.

### 3.3. Computational Details

All DFT calculations were performed in the Gaussian 09W [101] and Gaussview 5.0 [102] molecular package programs for the ground state and gas phase. At the first stage of the DFT study, the geometry of monomers was optimized. The two DFT methods used were the Becke’s three-parameter functional [103,104] and the gradient-corrected correlational functional proposed by Lee, Yang, and Parr (B3LYP) and its combination with the Coulomb attenuating method (CAM-B3LYP) [105,106]. For the optimized geometries, the molecular electrostatic potential (MEP) and the highest occupied molecular orbital (HOMO)—the lowest unoccupied molecular orbital (LUMO) analysis of betulin and sulfated betulin was made using the CAM-B3LYP/6-31 + G (d, p) level of theory. The FTIR and NMR theoretical analysis was made using the B3LYP/6-31 + G (d, p) level of theory.

## 4. Conclusions

A method for the sulfation of betulin with sulfamic acid in pyridine in the presence of a solid catalyst Amberlyst^®^15 is proposed. It has been shown that this catalyst remains stable in multicycle tests during sulfation of betulin with sulfamic acid in pyridine.

The resulting betulin sulfate was studied both by physicochemical methods, including FT-IR, UV-visible and NMR spectroscopy, X-ray diffraction analysis and TGA, and by DFT. The acidity constant of the acidic form of betulin sulfate has been studied. It is shown that the introduction of a sulfate group into the betulin molecule leads to the appearance of absorption bands in the FT-IR spectra characteristic of the S-O group. X-ray diffraction showed that sulfation leads to amorphization of the original betulin. It has been shown by UV-visible spectrophotometry that the introduction of a sulfate group into the betulin molecule leads to a decrease in the intensity of the peaks in the UV spectra. In addition, the average product of the first and second dissociation constants of the acidic form of betulin sulfate was determined.

Data on the most stable conformations and molecular electrostatic potential of betulin and betulin disulfate were obtained by the DFT method. The energy gaps for the original and sulfated betulin were found. It has been shown that betulin sulfate is more reactive than the original betulin. The DFT method was used to calculate the spectral characteristics of the original and sulfated betulin, which turned out to be in good agreement with the experimental data.

The developed sulfation method has great application potential, as it opens up new possibilities for separation, catalyst recycling, and reducing the cost of reagents.

## Figures and Tables

**Table 1:** Effect of conditions for the catalytic sulfation of betulin with sulfamic acid in pyridine on the sulfur content in the product.

No.	Number of Catalyst Cycles	Time, h	Sulfur Content, wt %
1	1	0.5	5,1
2	1	1.0	7,2
3	1	1.5	9.7
4	1	2.0	10.1
5	1	2.5	10.1
6	2	2.0	10.0
7	3	2.0	9.9
8	4	2.0	9.9

**Table 2:** Some electronic properties of betulin (BE) and betulin sulfate (BES) at the CAM-B3LYP and B3LYP/6-31+G (d, p) level of theory.

Parameter (eV)	BE		BES	
	CAM-B3LYP	B3LYP	CAM-B3LYP	B3LYP
E_HOMO_	−8.0636	−6.4970	−7.6140	−6.0592
E_LUMO_	2.0841	0.6027	−1.0534	−1.7704
Energy band gap 1 (∆E_1_ = E_LUMO_ − E_HOMO_)	10.1477	7.0997	6.5607	4.2888
E_HOMO-1_	−8.3901	−6.7860	−8.0834	−6.5468
E_LUMO+1_	2.8428	1.5094	−0.9562	−1.6803
Energy band gap 2 [∆E_2_ = (E_LUMO+1_) − (E_HOMO-1_)]	11.2329	8.2954	7.1272	4.8665
Chemical potential (μ)	−2.9897	−2.9471	−4.3337	−3.9148
Softness (ς)	0.1971	0.2817	0.3048	0.4663
Ionization energy (I)	8.0636	6.4970	7.6140	6.0592
Electron affinity (A)	−2.0841	−0.6027	1.0534	1.7704
Electronegativity (χ)	2.9897	2.9471	4.3337	3.9148
Chemical hardness (η)	5.0738	3.5499	3.2803	2.1444
Electrophilicity index (ω)	0.8808	1.2234	2.8626	3.5734
Maximum charge transfer index (ΔN_max_)	0.5892	0.8302	1.3211	1.8256
Nucleophilic index (N)	1.1353	0.8174	0.3493	0.2798
Optical softness (σ˳)	0.0985	0.1409	0.1524	0.2332

**Table 3:** Mulliken atomic charges of betulin (BE) and betulin sulfate (BES) at the CAM-B3LYP and B3LYP/6-31+G (d, p) levels of theory.

BE				BES			
Label	Symbol	CAM-B3LYP	B3LYP	Label	Symbol	CAM-B3LYP	B3LYP
1	C	−0.20309	−0.2021	1	C	−0.22973	−0.22872
2	C	0.18481	0.18906	2	C	0.18324	0.18934
3	C	0.01401	0.01816	3	C	0.00964	0.01468
4	C	−0.05888	−0.0508	4	C	−0.07512	−0.06891
5	C	0.05783	0.05865	5	C	0.05565	0.05635
6	C	−0.20435	−0.19927	6	C	−0.20148	−0.19788
7	C	−0.19031	−0.19141	7	C	−0.19045	−0.1918
8	C	−0.1909	−0.19068	8	C	−0.19249	−0.19222
9	C	0.04579	0.04836	9	C	0.04544	0.04774
10	C	−0.06271	−0.0556	10	C	−0.07765	−0.06991
11	C	0.07032	0.07734	11	C	0.06934	0.07627
12	C	−0.06643	−0.06291	12	C	−0.0649	−0.06222
13	C	−0.18767	−0.18994	13	C	−0.18787	−0.19012
14	C	−0.19296	−0.19412	14	C	−0.19225	−0.19329
15	C	−0.21532	−0.2128	15	C	−0.21202	−0.20973
16	C	−0.17216	−0.17377	16	C	−0.18315	−0.18554
17	C	0.01376	0.02268	17	C	−6.45E-4	0.00905
18	C	−0.07535	−0.07321	18	C	−0.07845	−0.07622
19	C	−0.19513	−0.19369	19	C	−0.1947	−0.1942
20	C	−0.18731	−0.18628	20	C	−0.19282	−0.1934
21	C	−0.11263	−0.11347	21	C	−0.11335	−0.11266
22	O	−0.56017	−0.55848	22	O	−0.57467	−0.58
23	C	−0.29325	−0.29311	23	C	−0.30288	−0.30325
24	C	−0.3145	−0.31645	24	C	−0.31418	−0.31685
25	C	−0.32162	−0.32217	25	C	−0.31836	−0.31921
26	C	0.05309	0.05169	26	C	0.03722	0.03911
27	C	−0.31074	−0.31217	27	C	−0.31306	−0.31459
28	C	−0.33367	−0.33536	28	C	−0.33572	−0.33768
29	C	0.15887	0.16828	29	C	0.16241	0.16996
30	C	−0.36417	−0.36923	30	C	−0.36498	−0.36949
31	C	−0.2996	−0.29698	31	C	−0.29878	−0.29611
32	O	−0.54748	−0.54509	32	O	−0.54583	−0.55022
33	H	0.1048	0.10177	33	H	0.12979	0.12677
34	H	0.09295	0.09169	34	H	0.09161	0.09019
35	H	0.08495	0.08254	35	H	0.09965	0.09586
36	H	0.05337	0.05213	36	H	0.13211	0.13113
37	H	0.10024	0.09727	37	H	0.08427	0.0821
38	H	0.0719	0.07075	38	H	0.13803	0.13532
39	H	0.0885	0.0889	39	H	0.07823	0.07888
40	H	0.09337	0.09139	40	H	0.08748	0.08604
41	H	0.08774	0.08523	41	H	0.07866	0.07653
42	H	0.08704	0.08641	42	H	0.09884	0.09788
43	H	0.06821	0.06817	43	H	0.10117	0.09971
44	H	0.08341	0.08274	44	H	0.07921	0.0789
45	H	0.09383	0.0913	45	H	0.0897	0.08761
46	H	0.08689	0.08867	46	H	0.09033	0.09176
47	H	0.08982	0.08987	47	H	0.08277	0.08326
48	H	0.08967	0.08751	48	H	0.09089	0.08839
49	H	0.09365	0.09198	49	H	0.08868	0.08699
50	H	0.08919	0.08727	50	H	0.09186	0.0901
51	H	0.09946	0.09543	51	H	0.1076	0.10509
52	H	0.08699	0.08781	52	H	0.08374	0.08357
53	H	0.08231	0.0818	53	H	0.08349	0.08281
54	H	0.06581	0.06409	54	H	0.12012	0.11787
55	H	0.09886	0.09787	55	H	0.09005	0.08945
56	H	0.1011	0.10052	56	H	0.11287	0.11204
57	H	0.09352	0.09118	57	H	0.08921	0.08717
58	H	0.08797	0.08677	58	H	0.08784	0.08778
59	H	0.30411	0.30184	59	H	0.1285	0.12782
60	H	0.10399	0.10187	60	H	0.09061	0.08926
61	H	0.09188	0.09124	61	H	0.09559	0.09434
62	H	0.09807	0.09677	62	H	0.0856	0.08599
63	H	0.0944	0.09438	63	H	0.09401	0.09461
64	H	0.09969	0.09982	64	H	0.09462	0.09426
65	H	0.09838	0.09805	65	H	0.0911	0.09123
66	H	0.09449	0.0942	66	H	0.09448	0.09386
67	H	0.10248	0.10202	67	H	0.09695	0.09611
68	H	0.09944	0.09872	68	H	0.1333	0.13009
69	H	0.0819	0.08019	69	H	0.11729	0.11281
70	H	0.1016	0.10022	70	H	0.10554	0.10448
71	H	0.10214	0.10139	71	H	0.09637	0.09664
72	H	0.09582	0.0964	72	H	0.09323	0.09188
73	H	0.09797	0.09658	73	H	0.10952	0.1082
74	H	0.09717	0.09619	74	H	0.09666	0.09747
75	H	0.09912	0.09985	75	H	0.10339	0.10164
76	H	0.10177	0.1	76	H	0.11443	0.11385
77	H	0.11392	0.11365	77	H	0.10407	0.1031
78	H	0.10872	0.10765	78	H	0.11323	0.1131
79	H	0.11357	0.11327	79	H	0.09275	0.09116
80	H	0.09079	0.08897	80	H	0.08375	0.0818
81	H	0.08773	0.08596	81	S	1.40967	1.41359
82	H	0.30721	0.30457	82	O	−0.62093	−0.6217
-	-	-	-	83	O	−0.52194	−0.52206
-	-	-	-	84	O	−0.64718	−0.64532
-	-	-	-	85	Na	0.59859	0.60197
-	-	-	-	86	S	1.44977	1.45217
-	-	-	-	87	O	−0.53987	−0.53873
-	-	-	-	88	O	−0.62287	−0.62426
-	-	-	-	89	O	−0.65891	−0.65632
-	-	-	-	90	Na	0.60311	0.60553

**Table 4:** Some important FTIR data (cm^−1^) calculated at B3LYP/6-31 + G (d, p) for betulin (BE) and betulin sulfate (BES) and scaled by a factor of 0.9608.

BE		BES	
O-H	3671 and 3670	C-H (CH_2_=)	3107 and 3032
C-H (CH_2_=)	3107 and 3032	C-H	3033–2901
C-H	3038–2835	C-H (=C-CH_3_)	2998 and 2905
C-H (=C-CH3)	3002 and 2906	C=C	1658
C-H (CH2-OH)	2954 and 2870	O-S	1227, 1209, 1074, 1068, 941, 931
CH (CH-OH)	2940 and 2925	CH_2_ (O-)	2984, 1474, 1210
C=C	1657	Na-O	307 and 295

**Table 5:** ^1^HNMR and ^13^CNMR data for betulin (BE) and sulfated betulin (BES) calculated at B3LYP/6-31 + G (d, p).

BE				BES			
Hydrogen Atom	Chemical Shift (ppm)	Carbon Atom	Chemical Shift (ppm)	Hydrogen Atom	Chemical Shift (ppm)	Carbon Atom	Chemical Shift (ppm)
80-H	5.1189	29-C	140.0163	79-H	5.156	29-C	140.1273
81-H	5.0522	31-C	93.0736	80-H	5.0961	31-C	93.0158
69-H	3.8844	2-C	67.3998	68-H	4.2101	2-C	80.5108
35-H	3.4006	26-C	61.5732	35-H	4.1063	26-C	63.9693
70-H	3.2142	10-C	44.2637	69-H	3.644	10-C	42.9424
58-H	3.0378	4-C	42.8496	58-H	3.1113	18-C	41.8903
44-H	2.2612	18-C	41.2406	36-H	2.7828	21-C	40.439
34-H	2.2327	17-C	40.3549	33-H	2.6308	4-C	40.0096
57-H	2.2152	21-C	40.2103	57-H	2.311	17-C	38.9887
79-H	2.0347	9-C	38.2528	44-H	2.2933	9-C	38.1393
55-H	2.0335	11-C	36.727	34-H	2.124	11-C	36.4112
77-H	1.9339	3-C	32.7851	38-H	2.1138	3-C	32.9181
49-H	1.9317	5-C	32.6781	78-H	2.0739	5-C	32.3275
51-H	1.8729	12-C	29.4602	55-H	2.0641	12-C	29.5149
39-H	1.8035	20-C	26.4429	76-H	1.9848	20-C	26.7763
53-H	1.796	6-C	25.4542	54-H	1.9815	6-C	25.9807
78-H	1.7151	8-C	24.085	49-H	1.9654	8-C	23.9728
47-H	1.6804	16-C	22.1573	53-H	1.9604	19-C	22.9769
43-H	1.6769	19-C	22.0667	43-H	1.944	16-C	21.3784
42-H	1.6543	15-C	20.998	51-H	1.844	15-C	20.8974
48-H	1.6326	23-C	20.0705	39-H	1.7884	23-C	18.9603
37-H	1.6259	13-C	18.8465	77-H	1.7473	13-C	18.6958
46-H	1.6253	1-C	17.938	42-H	1.7136	30-C	16.8118
40-H	1.582	30-C	16.8562	47-H	1.6841	1-C	14.6996
33-H	1.563	14-C	14.6112	46-H	1.6692	27-C	14.5464
41-H	1.5611	27-C	13.6592	48-H	1.654	14-C	14.3916
74-H	1.5597	28-C	13.0177	40-H	1.6504	28-C	12.8152
54-H	1.5306	7-C	12.1492	73-H	1.6266	7-C	12.3059
45-H	1.5145	25-C	7.8876	45-H	1.5462	24-C	7.7267
68-H	1.494	24-C	7.6367	41-H	1.5179	25-C	7.5684
52-H	1.4371	-	-	52-H	1.5156	-	-
56-H	1.3537	-	-	37-H	1.4956	-	-
59-H	1.3506	-	-	56-H	1.4865	-	-
50-H	1.2945	-	-	67-H	1.4568	-	-
76-H	1.2901	-	-	75-H	1.3469	-	-
72-H	1.2891	-	-	59-H	1.3319	-	-
36-H	1.2623	-	-	50-H	1.3168	-	-
60-H	1.2384	-	-	60-H	1.2996	-	-
61-H	1.196	-	-	71-H	1.2321	-	-
38-H	1.1732	-	-	70-H	1.1811	-	-
71-H	1.1562	-	-	63-H	1.1485	-	-
64-H	1.1243	-	-	65-H	1.1308	-	-
75-H	1.1019	-	-	74-H	1.1244	-	-
66-H	1.0916	-	-	64-H	0.965	-	-
63-H	1.0154	-	-	62-H	0.9579	-	-
65-H	0.9691	-	-	61-H	0.9354	-	-
67-H	0.8833	-	-	66-H	0.8736	-	-
82-H	0.8301	-	-	72-H	0.5335	-	-
62-H	0.7253	-	-	-	-	-	-
73-H	0.6054	-	-	-	-	-	-

## Data Availability

Data sharing not applicable to this article as no datasets were generated or analysed during the current study. All the data generated during this study are included in this article.

## Sample Availability

Samples of the compounds are available from the authors.
